# Comparative Study of Pd–Ni Bimetallic Catalysts Supported on UiO-66 and UiO-66-NH_2_ in Selective 1,3-Butadiene Hydrogenation

**DOI:** 10.3390/nano12091484

**Published:** 2022-04-27

**Authors:** Lili Liu, Lei Yu, Xiaojing Zhou, Chunling Xin, Songyuan Sun, Zhidong Liu, Jinyu Zhang, Ying Liu, Xishi Tai

**Affiliations:** 1 School of Chemistry & Chemical Engineering and Environmental Engineering, Weifang University, Weifang 261061, China; liulili122@wfu.edu.cn (L.L.); jimoyulei@163.com (L.Y.); zhouxiaojing105@wfu.edu.cn (X.Z.); 20150015@wfu.edu.cn (C.X.); ssy1210661650@foxmail.com (S.S.); liuzhidong0518@foxmail.com (Z.L.); 2 Shandong Huazhiyuan Testing Co., Ltd., Weifang 261061, China; ziyjinyu@163.com

**Keywords:** Pd–Ni bimetallic catalysts, UiO-66, UiO-66-NH_2_, 1,3-butadiene hydrogenation

## Abstract

Selective hydrogenation of 1,3-butadiene (BD) is regarded as the most promising route for removing BD from butene streams. Bimetallic Pd–Ni catalysts with changed Pd/Ni molar ratios and monometallic Pd catalysts were synthesized using two differently structured metal-organic framework supports: UiO-66 and UiO-66-NH_2_. The effects of the structure of support and the molar ratio of Pd/Ni on the catalytic property of selective BD hydrogenation were studied. The Pd–Ni bimetallic supported catalysts, PdNi/UiO-66 (1:1) and PdNi/UiO-66-NH_2_ (1:1), exhibited fine catalytic property at low temperature. Compared with UiO-66, UiO-66-NH_2_ with a certain number of alkaline sites could reduce the catalytic activity for the BD hydrogenation reaction. However, the alkaline environment of UiO-66-NH_2_ is helpful to improve the butene selectivity. PdNi/UiO-66-NH_2_ (1:1) catalyst presented better stability than PdNi/UiO-66 (1:1) under the reaction conditions, caused by the strong interaction between the –NH_2_ groups of UiO-66-NH_2_ and PdNi NPs. Moreover, the PdNi/UiO-66-NH_2_ (1:1) catalyst presented good reproducibility in the hydrogenation of BD. These findings afford a beneficial guidance for the design and preparation of efficient catalysts for selective BD hydrogenation.

## 1. Introduction

Butenes are key raw materials for polymer synthesis [1,2,3]. However, the butene streams produced from petroleum cracking contains small amounts of 1,3-butadiene (BD), which must be removed in the subsequent polymerization processing because it could cause catalyst poisoning and product quality degradation in downstream processes [4,5]. Selective BD hydrogenation is regarded as the most promising route for removing BD from butene streams [6,7]. In the past few decades, various catalysts, such as Au [8], Pt [9], Cu [10], Co [11], Pd [12], Pd–Au [13,14], Pd–Cu [1], Pd–Ag [15,16], Pd–Ni [17], Au–Ni [18], and Pt–Cu [19], have been developed and used in BD hydrogenation. Among them, bimetallic supported catalysts displayed excellent catalytic property for BD hydrogenation due to the synergistic effect between metals [17,18]. Lucci et al. [19] found that bimetallic PtCu/Al_2_O_3_ catalyst displayed 99% butene selectivity at 100% BD conversion. Lu et al. [15] showed that AgPd/g-Al_2_O_3_ catalysts displayed excellent BD conversion (98.2%) and butene selectivity (88.1%) in BD hydrogenation. Pattamakomsan et al. [6] reported that PdSn/Al_2_O_3_ catalysts not only have high 1-butene selectivity, but could also avoid deep hydrogenation and isomerization.

The support has a considerable effect on the catalytic performance for the selective BD hydrogenation reaction [20,21,22,23,24,25]. The interactions among metal NPs and supports, charging transfers, and support acidity play important roles in the catalytic activity and product selectivity in BD hydrogenation [20,21]. Huang et al. [20] found that Pd1/graphene displayed higher activity than Pd1/C_3_N_4_ in the hydrogenation of BD. Hou et al. [21] investigated the impact of oxide supports on the BD hydrogenation over Pd–Ni bimetallic catalysts. They found that the chemical property of oxide support had no effect on activity of the catalyst, but it did have a significant effect on 1-butene selectivity because of the tough metal–support interaction and oxygen defects [21]. Decarolis et al. [22] also reported that the support type had a great influence on the catalytic property in BD selective hydrogenation.

Recently, the metal atomic ratio of bimetallic catalysts has been observed to affect the catalytic performance [7,26,27,28,29,30]. Méndez et al. [29] found that adding Ni to the Pd based catalysts decreased the hydrogenation activity (BD conversion) and improved the selectivity of the total butene at moderate BD conversions. The 1NiPd/Al_2_O_3_ catalyst (Pd = 0.5 wt%, Ni/Pd molar ratio of 1) exhibited improved catalytic performance for the BD selective hydrogenation [29]. Lonergan et al. [26] studied the effects of Pt:Ni atomic ratio of PtNi/γ-Al_2_O_3_ on the hydrogenation of BD. They observed that the catalysts having a lower Pt:Ni atomic ratio presented elevated catalytic activity [31]. Odoom-Wubah et al. [7] reported that Pd−Cu_0.06_/Mn_2_O_3_-SI (SI = sol-immobilization) showed an effective balance between BD conversion and butene selectivity at room temperature. The catalyst Au(2)Pd(1)/MIL-101(Cr) displayed the best hydrogenation performance in the selective BD hydrogenation [31].

Metal–organic frameworks (MOFs) are greatly regular porous coordination polymers that have been widely used as a support in heterogeneous catalysts due to their large pore volumes and uniform cavities [32,33,34]. Bimetallic nanoparticle (NP) catalysts supported on MOF have been recently reported [35,36,37,38]. For instance, a ZIF-8 supported core-shell Ag@Au bimetallic catalyst (Ag@Au/ZIF-8) exhibited high conversion and selectivity of benzaldehyde in benzyl alcohol oxidation [38]. Au-Pd/MIL-101(Cr) NP catalysts displayed improved catalytic activity for BD hydrogenation [31]. Ni–Pt NPs supported on MIL-101-NH_2_ displayed a good activity with a 137 h^−1^ turnover frequency (TOF) when hydrazine hydrate completely converted into hydrogen at 25 °C [36]. Ten et al. [39] also found that AgPd/UiO-66 catalysts showed good catalytic activity in propylene glycol oxidation due to the synergistic interaction between the components in Ag–Pd alloyed NPs. They also found that the catalytic activity was largely dependent on the preparation process and the properties of the metal precursors [39]. The AgPd/UiO-66 catalyst prepared by the double solvent method with aqueous nitrate salts as precursors and N_2_H_4_ reduction displayed the highest propylene glycol conversion [39].

Among the various reported MOFs, the UiO-66 series is an outstanding one for its high surface area, as well as its good chemical resistance to organic solvents and water and high thermal stability (>500 °C) [40,41]. In addition, the UiO-66 derivatives series, i.e., UiO-66-OH, UiO-66-COOH, UiO-66-NH_2_, and UiO-66-NO_2_, can be obtained through ligand functionalization [42,43]. Among them, UiO-66 and UiO-66-NH_2_ have recently attracted great interest and have been widely used in adsorption and separation [44,45], luminescent sensing [46], antibacterial and antiviral [47], and catalytic reactions [48]. Especially, UiO-66 and UiO-66-NH_2_ have been used as a support for metal NPs in catalyzing cinnamaldehyde hydrogenation [48], acetic acid hydrogenation [49], phenol hydrogenation [40], and CO_2_ hydrogenation [50]. However, there are few studies about the effect of UiO-66 and UiO-66-NH_2_ on the catalytic properties in BD hydrogenation. It has been reported that the basic and acidic nature of the support could markedly affect the general butene distribution and selectivity of 1-butene [5,51]. Acidic supports were less selective to the total butenes and inhibited the 1-butene selectivity [5,51]. In the present work, the catalytic property of UiO-66 and UiO-66-NH_2_-supported Pd–Ni bimetallic NPs or Pd NPs in the selective BD hydrogenation reaction was compared and investigated. The effect of support structures and the Pd/Ni molar ratio of the catalysts PdNi/UiO-66 and PdNi/UiO-66-NH_2_ on the catalytic activity and selectivity were studied. PdNi/UiO-66 displayed higher activity than PdNi/UiO-66-NH_2_ in BD hydrogenation. However, PdNi/UiO-66-NH_2_ displayed improved selectivity to total butenes and stability due to the interaction among PdNi NPs and support. Furthermore, the Pd/Ni molar ratio of bimetallic Pd–Ni catalysts had a significant effect on the activity and product selectivity.

## 2. Experimental Section

### 2.1. Synthesis of UiO-66-NH_2_ and UiO-66

The UiO-66-NH_2_ crystal was prepared via the solvothermal method [52]. ZrCl_4_ (2.40 mmol, 0.56 g) was melted into N,N’-dimethylformamide (DMF, 9 mL) under intense stirring. The 2-aminoterephthalic acid (NH_2_-BDC, 5.10 mmol, 0.92 g) and benzoic acid (72.06 mmol, 8.80 g) were melted into 20 mL DMF. Afterwards, the DMF solution of ZrCl_4_ was added into the solution of NH_2_-BDC and benzoic acid. After stirring for 1 h, the homogenized solution was heated at 180 °C in a 100 mL Teflon autoclave for 24 h in a drying oven. The light-yellow powder was collected by centrifugation at 3800 rpm for 20 min. The powder was washed four times with DMF and methanol, respectively. Finally, it was dried for 24 h in a vacuum oven at 60 °C. UiO-66 was also prepared via the solvothermal method by using the same procedure, except using terephthalic acid (BDC) instead of NH_2_-BDC.

### 2.2. Synthesis of Pd–Ni Bimetallic Catalysts

Pd–Ni bimetallic catalysts were synthesized via the impregnation method using Ni(NO_3_)_2_·6H_2_O and Pd(CH_3_COO)_2_ as precursors [31]. First, Ni(NO_3_)_2_·6H_2_O (0.086 mmol, 25.0 mg) and Pd(CH_3_COO)_2_ (0.086 mmol, 19.2 mg) were dissolved into 1 mL absolute ethanol. Second, the mixture of Ni(NO_3_)_2_·6H_2_O and Pd(CH_3_COO)_2_ was added to UiO-66-NH_2_ (0.3 g) drop by drop. The samples were ultrasonically treated at 20 °C for 1 h and then aged in the refrigerator at 4 °C for 24 h, followed by drying in a vacuum oven at 50 °C for 2 h. Finally, they were reduced in the H_2_ stream (12.0 mL/min) at 50 °C for 2 h and labeled as PdNi/UiO-66-NH_2_ (1:1). PdNi/UiO-66-NH_2_ catalysts with Pd:Ni molar ratios of 1:3, 1:2, 1:1, 2:1, and 3:1 were synthesized for investigating the effects of Pd:Ni molar ratio on the activity and selectivity of BD hydrogenation, with a total metal (Pd and Ni) content of ca. 4.0 wt.% (Table S1). A series of PdNi/UiO-66 catalysts with Pd:Ni molar ratios of 1:3, 1:2, 1:1, 2:1, and 3:1 was also prepared via the impregnation method (Table S1). The Pd/UiO-66 and Pd/UiO-66-NH_2_ catalysts were also synthesized via the impregnation method using Pd(CH_3_COO)_2_ as precursor (Table S1).

### 2.3. Catalytic Activity Measurement

The catalytic property of Pd–Ni NP and Pd NP supported catalysts was assessed using a continuous flowing system with a 6 mm inner diameter of quartz fixed-bed reactor under atmospheric pressure. Initially, 5 mg of fine powder catalyst was diluted with 0.5 g quartz sand (25–40 mesh), then placed at the center of the quartz reactor. The reactants, H_2_ (6.5 mL/min) and BD/N_2_ (1.0 vol.%, 20 mL/min), passed through the fixed catalyst bed with a space velocity of 318,000 mL/(h·g_cat_) at 40–110 °C. The high space velocity can eliminate significant mass-transfer limitation during the hydrogenation of BD. The reactor effluent was measured online using the Al_2_O_3_ capillary column.

## 3. Results and Discussion

### 3.1. Characterization of Catalysts

**Figure 1:**
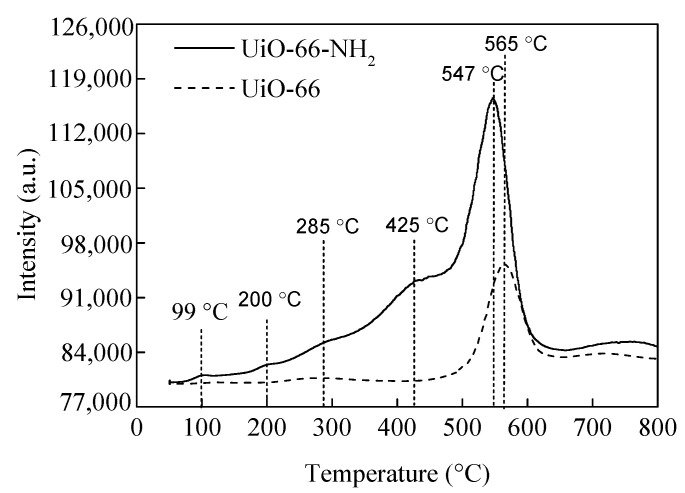
CO_2_-TPD profiles for UiO-66-NH_2_ and UiO-66.

**Figure 2:**
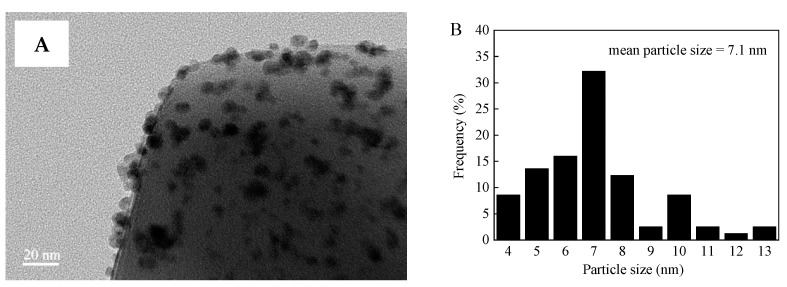

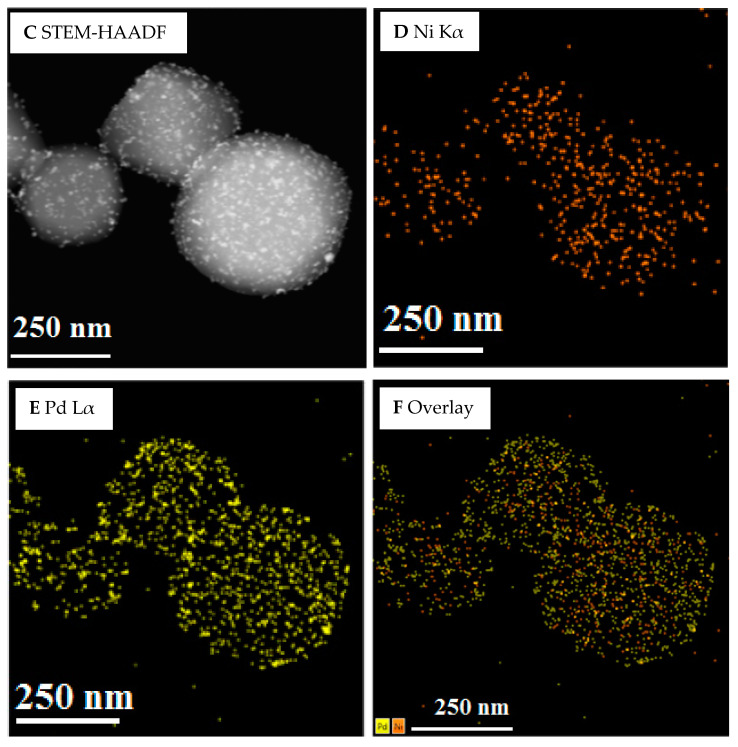
(**A**) TEM image, (**B**) PdNi size distribution, (**C**) STEM-HAADF image, and (**D**–**F**) EDS elemental mapping images of PdNi/UiO-66 (1:1).

**Figure 3:**
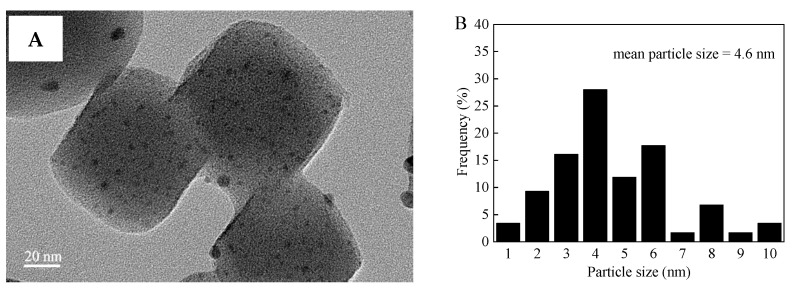

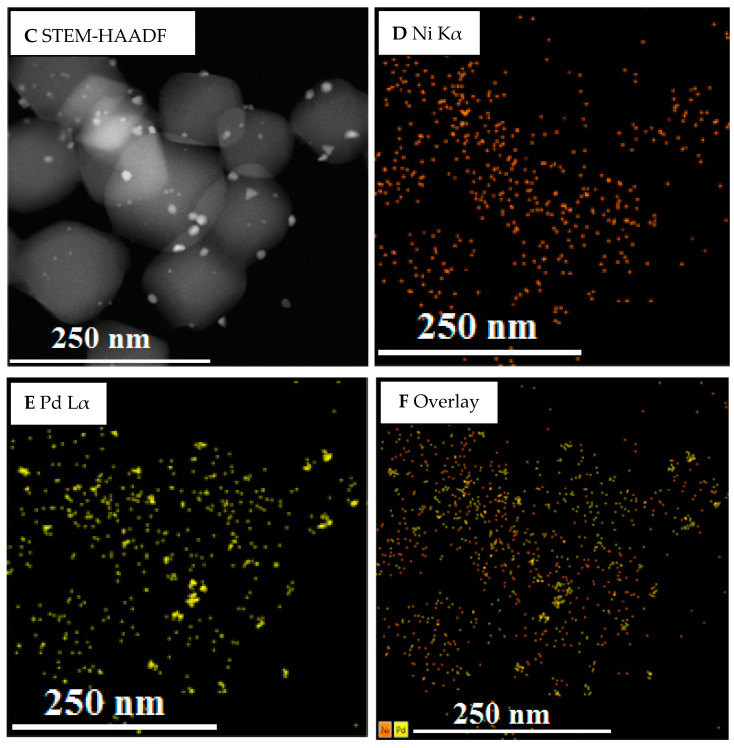
(**A**) TEM image, (**B**) PdNi size distribution, (**C**) STEM-HAADF image, and (**D**–**F**) EDS elemental mapping images of PdNi/UiO-66-NH_2_ (1:1).

The MOF supports and Pd–Ni bimetallic catalysts were investigated in detail by CO_2_-TPD, PXRD, N_2_ adsorption-desorption, TEM, EDS, XPS, and ICP-OES. Figure 1 displays the CO_2_-TPD profiles of UiO-66 and UiO-66-NH_2_. For UiO-66-NH_2_, five peaks were observed when the desorption temperature was below 800 °C. The peaks located at 99 °C and 425 °C corresponded to weak basic sites and strong basic sites, respectively, while those peaks at 200 °C and 285 °C were ascribed to medium basic sites [53,54,55,56]. In addition, a strong peak at 547 °C is observed, originating from the decomposition of UiO-66-NH_2_ [53,54,55,56]. However, only one CO_2_ desorption peak (565 °C) appeared on the UiO-66 CO_2_-TPD profile, which is attributed to the decomposition of UiO-66. This phenomenon was induced by –NH_2_ groups present on UiO-66-NH_2_ [55]. CO_2_-TPD characterization displayed a certain number of alkaline sites on UiO-66-NH_2_, whereas no alkaline sites were found on UiO-66. The position and intense PXRD peaks of UiO-66 and UiO-66-NH_2_ were in good agreement with those reported in literature, illustrating that the samples were synthesized successfully (Figure S1) [42,44,46]. Furthermore, the PXRD diffraction peaks of UiO-66 and UiO-66-NH_2_ were the same because both have similar ligands (Figure S1) [44]. The peak position and intensity of PdNi/UiO-66-NH_2_ (1:1), Pd/UiO-66-NH_2_, PdNi/UiO-66 (1:1), and Pd/UiO-66 were similar to those of undeposited UiO-66 and UiO-66-NH_2_, thus proving that the structures of UiO-66-NH_2_ and UiO-66 were well maintained after the deposition of Pd–Ni NPs or Pd NPs (Figure S1). No characteristic peaks of Pd–Ni NPs, Pd NPs, and Ni NPs were observed in the PXRD patterns of PdNi/UiO-66-NH_2_ (1:1) and PdNi/UiO-66 (1:1) due to the low Pd and Ni contents in the catalyst [57,58]. The adsorption/desorption isotherms of N_2_ and textural properties on different samples are displayed in Figure S2 and Table 1. The sorption isotherms exhibited the type IV curves, suggesting that UiO-66, PdNi/UiO-66 (1:1), UiO-66-NH_2_, and PdNi/UiO-66-NH_2_ (1:1) possessed a mesoporous structure (Figure S2A). The BET surface areas of UiO-66, PdNi/UiO-66 (1:1), UiO-66-NH_2_, and PdNi/UiO-66-NH_2_ (1:1) were 1467, 1292, 1223, and 851 m^2^/g, respectively. The pore volume of UiO-66, PdNi/UiO-66 (1:1), UiO-66-NH_2_, and PdNi/UiO-66-NH_2_ (1:1) were 0.75 cm^3^/g, 0.70 cm^3^/g, 1.02 cm^3^/g, and 0.81 cm^3^/g, respectively. The BET surface area of UiO-66-NH_2_ was smaller than UiO-66 because of the involvement of –NH_2_ in the pores [40,42]. After the deposition of PdNi NPs on UiO-66 and UiO-66-NH_2_, the pore volume and BET surface area decreased significantly, indicating that the addition of PdNi NPs resulted in a decrease in the pore volume and BET surface area of the UiO-66 and UiO-66-NH_2_ [40]. The reduction in BET surface areas and pore volume is ascribed to the PdNi NPs occupying or blocking the pores of UiO-66 and UiO-66-NH_2_ [59]. The UiO-66 and PdNi/UiO-66 (1:1) displayed a similar pore size distribution, and pore sizes are mainly distributed in 0.59, 0.82, 1.05, 1.25, and 3.21 nm. Moreover, UiO-66, after depositing PdNi NPs, presented a new pore size at 0.45 nm, which may be due to the involvement of PdNi NPs in the pores of UiO-66 (Figure S2B). The sample of UiO-66-NH_2_ and PdNi/UiO-66-NH_2_ (1:1) also presented similar pore size distributions, and a new pore size appeared at 0.35 nm (Figure S2C). However, the mean pore diameter increased after depositing PdNi NPs. The mean pore diameter of UiO-66, PdNi/UiO-66 (1:1), UiO-66-NH_2_, and PdNi/UiO-66-NH_2_ (1:1) was 2.1, 2.2, 3.3, and 3.8 nm, respectively. The increase of mean pore size of UiO-66 and UiO-66-NH_2_ after depositing PdNi NPs was mainly because PdNi NPs could block the pores of UiO-66 and UiO-66-NH_2_ [40]. TEM images of PdNi/UiO-66-NH_2_ (1:1), Pd/UiO-66-NH_2_, PdNi/UiO-66 (1:1), and Pd/UiO-66 revealed that the PdNi NPs or Pd NPs substantially dispersed on the surface of the UiO-66 and UiO-66-NH_2_ support, with mean particle sizes of 4.6 nm, 7.7 nm, 7.1 nm, and 14.6 nm, respectively (Figure 2A,B, Figure 3A,B and Figure S3). The PdNi NPs or Pd NPs deposited on UiO-66-NH_2_ are smaller than those deposited on UiO-66. Moreover, the particle size of PdNi NPs is smaller than that of Pd NPs when deposited on the same support. This may be because the amine group of UiO-66-NH_2_ can act as an anchoring group to stabilize PdNi NPs or Pd NPs. In addition, the interaction between Pd and Ni could effectively prevent the aggregation of PdNi NPs. The EDS mapping analysis of PdNi/UiO-66-NH_2_ (1:1) and PdNi/UiO-66 (1:1) displayed that Pd and Ni were evenly distributed in the catalysts (Figure 2C–F and Figure 3C–F) [31,60]. As shown in Figure 2F and Figure 3F, the overlap of Pd and Ni signal is not perfect, indicating that individual monometallic Pd and Ni NPs are formed on the surface of UiO-66 and UiO-66-NH_2_ [39]. XPS measurement displayed that the Pd and Ni in the PdNi/UiO-66 (1:1) and PdNi/UiO-66-NH_2_ (1:1) catalysts existed as metallic Pd (Pd^0^) and oxidation-state Ni (NiO) (Figure S4) [31,60,61]. The structure of the MOF support has no influence on the Pd and Ni valence state of PdNi/UiO-66-NH_2_ (1:1) and PdNi/UiO-66 (1:1).

### 3.2. Catalytic Performance

**Figure 4:**
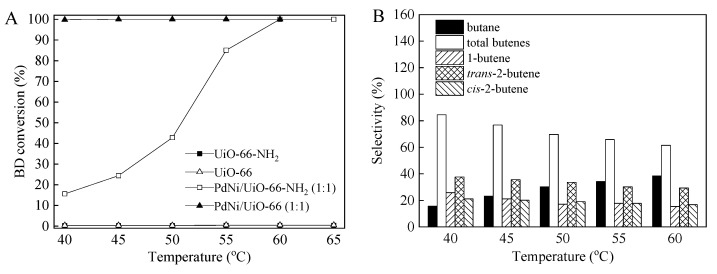

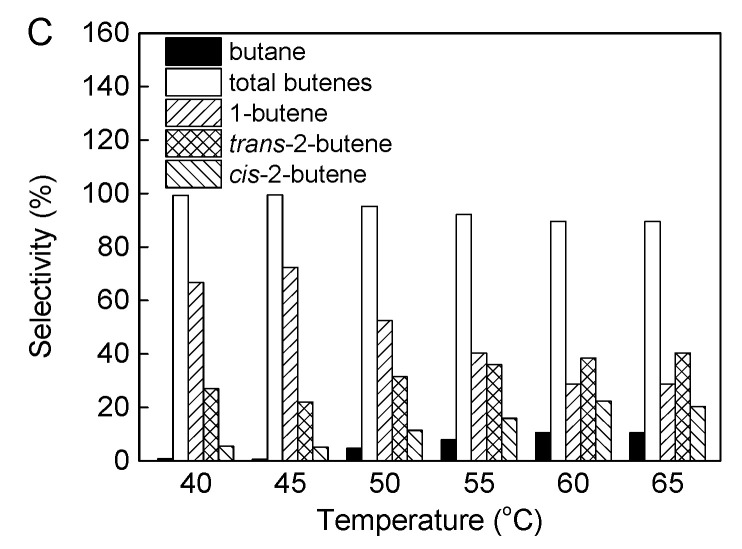
(**A**) BD conversions for UiO-66, UiO-66-NH_2_, PdNi/UiO-66 (1:1) and PdNi/UiO-66-NH_2_ (1:1); (**B**,**C**) Product selectivities for PdNi/UiO-66 (1:1) (**B**) and PdNi/UiO-66-NH_2_ (1:1) (**C**) as a function of temperature (reaction conditions: 5 mg of catalyst, 6.5 mL/min of H_2_ flow rate, 20 mL/min of 1.0 vol.%BD/N_2_ flow rate. BD conversion and product selectivities given in (**A**–**C**) were stable data at reaction temperature).

UiO-66 and UiO-66-NH_2_ MOFs were chosen to compare the influence of the support on the catalytic activity and selectivity for the selective hydrogenation of BD. The Pd–Ni bimetallic catalysts supported on UiO-66 and UiO-66-NH_2_ were investigated in the BD hydrogenation reaction. The evolution of BD conversion and selectivity for PdNi/UiO-66 (1:1) and PdNi/UiO-66-NH_2_ (1:1) in BD hydrogenation as a function of the reaction temperature (40–65 °C) are presented in Figure 4. The conversion of BD for UiO-66 and UiO-66-NH_2_ supports are also illustrated in Figure 4. The supports of UiO-66 and UiO-66-NH_2_ presented a low BD conversion of less than 1% at 40–65 °C (Figure 4A). The bimetallic PdNi/UiO-66 (1:1) catalysts exhibited higher catalytic activity than PdNi/UiO-66-NH_2_ (1:1) (Figure 4A). A BD conversion of 99.8% was achieved on PdNi/UiO-66 (1:1) at 40 °C, and then it remained constant (Figure 4A). The BD conversion was only 15.6% for PdNi/UiO-66-NH_2_ (1:1) at 40 °C. Then, it gradually increased with the increase in the reaction temperature, up to 100% at 60 °C (Figure 4A). The products of BD hydrogenation mainly include butane, 1-butene, *trans*-2-butene, and *cis*-2-butene, among which 1-butene, *trans*-2-butene, and *cis*-2-butene are the ideal products and butane is the by-product. For the PdNi/UiO-66 (1:1) catalyst, the selectivities to total butenes ranged between 61.5% and 84.5% and reduced when the temperature was increased to 60 °C from 40 °C (Figure 4B). A continuous reduction in the selectivity to 1-butene, *cis*-2-butene, and *trans*-2-butene was detected with the raise in temperature, whereas the selectivity to butane increased, suggesting that butenes were hydrogenated to produce butane on PdNi/UiO-66 (1:1) in the BD hydrogenation reaction (Figure 4B). Compared with PdNi/UiO-66 (1:1), PdNi/UiO-66-NH_2_ (1:1) displayed different selectivities of butane and total butenes. For the PdNi/UiO-66-NH_2_ (1:1) catalyst, the selectivity to butane and total butenes remained unchanged at low reaction temperatures (40 °C and 45 °C) (Figure 4C). However, the selectivity to total butenes was reduced slightly by raising temperature from 50 °C to 65 °C, whereas the selectivity to butane increased slightly (Figure 4C). The selectivities to butane and total butenes were 11.5% and 88.5%, 10.5% and 89.5%, 7.8% and 92.2%, 4.7% and 95.3%, 0.5% and 99.5%, and 0.7% and 99.3% at 65 °C, 60 °C, 55 °C, 50°C, 45 °C, and 40°C, respectively. The selectivity to total butenes (88.5–99.5%) on PdNi/UiO-66-NH_2_ (1:1) was higher than that on PdNi/UiO-66 (1:1) (61.5–84.5%). The selectivity to 1-butene, *cis*-2-butene, and *trans*-2-butene were similar on PdNi/UiO-66-NH_2_ (1:1) catalysts at low temperatures (40 °C and 45 °C), indicating that no isomerization was detected during BD hydrogenation at low temperature (Figure 4C) [21,31]. At high reaction temperatures (50–65 °C), the selectivity of 1-butene reduced with the raise of temperature, while that of *cis*-2-butene and *trans*-2-butene improved with the raise of temperature (Figure 4C). This phenomenon indicated that 1-butene isomerized to *cis*-2-butene and *trans*-2-butene during BD hydrogenation at high reaction temperature [21,59]. The activity and selectivity of the Pd–Ni-supported catalysts were influenced critically by the structure of the MOF support. TEM characterization presented that the mean particle size of PdNi/UiO-66-NH_2_ (1:1) (4.6 nm) is smaller than PdNi/UiO-66 (7.1 nm) due to the amine groups of UiO-66-NH_2_. According to previously reported studies, the size of nanoparticles is the primary factor affecting the activity and selectivity for the BD hydrogenation reaction, and the catalyst with smallest NP size presented higher BD conversion [31]. However, the Pd-Ni NPs supported on UiO-66 was more active than that supported on UiO-66-NH_2_. This could be due to the influence of MOF support structure. Compared with UiO-66, UiO-66-NH_2_ with a certain number of alkaline sites could inhibit the catalytic activity for BD hydrogenation reaction. On the contrary, the alkaline environment of UiO-66-NH_2_ is helpful to improve the butene selectivity. The selectivity of the PdNi/UiO-66 (1:1) to total produced butenes was approximately 84.5% at 99.8% BD conversion at 40 °C, while that of the PdNi/UiO-66-NH_2_ (1:1) catalyst was 89.5% at 100% BD conversion at 60 °C (Figure 4B,C). These results are consistent with previous studies [40,62,63]. Zhang et al. [63] reported that Pd/Al_2_O_3_-TiO_2_ catalyst modified with alkaline additive displayed better butene selectivity for the BD hydrogenation. Guan et al. [40] found that the structural differences between Pd-UiO-66-NH_2_ and Pd-UiO-66 were the reasons for the different activities and the product selectivities of the two catalysts in the phenol hydrogenation. Liang et al. [62] reported that the photocatalytic activity of ZnTCPc/UIO-66(NH_2_) with a stable covalent bond for methylene blue degradation was higher than that of ZnTCPc/UIO-66.

In order to evaluate the catalytic performance of the synthesized catalyst in the BD hydrogenation, the BD conversion and selectivity to total butenes on different supported Pd–Ni bimetallic catalysts were compared. The results are summarized in Table 2. PdNi/γ-Al_2_O_3_ catalyst displayed good catalytic property, i.e., 90% conversion of BD and 80% selectivity of butenes at 70 °C [21]. The BD conversion and butene selectivity reached 85% and 100% at 70 °C on PdNi/SiO_2_ catalyst [21]. Méndez et al. reported that 1NiPd/Al_2_O_3_ afforded excellent catalytic activity with a BD conversion and butene selectivity of 80% and 99.8% at 40 °C, respectively [29]. A complete BD conversion (100%) was achieved on 0.91%Pd1.51%Ni/γ-Al_2_O_3_ catalyst at 87 °C [64]. Based on the above results, our PdNi/UiO-66 (1:1) and PdNi/UiO-66-NH_2_ (1:1) catalysts display much higher catalyst activity and butene selectivity than PdNi/γ-Al_2_O_3_ and 0.91%Pd1.51%Ni/γ-Al_2_O_3_ for BD hydrogenation. Although the selectivity of PdNi/UiO-66 (1:1) and PdNi/UiO-66-NH_2_ (1:1) is lower than that of PdNi/SiO_2_, they display higher BD conversions at lower reaction temperatures. The PdNi/UiO-66 (1:1) catalyst shows higher BD conversions than 1NiPd/Al_2_O_3_ catalyst at 40 °C, but its selectivity of butene is smaller than 1NiPd/Al_2_O_3_. Although the BD conversion and butene selectivity of PdNi/UiO-66-NH_2_ (1:1) are less than that of 1NiPd/Al_2_O_3_, the excellent stability (16 h) of the catalyst shows the advantage of using UiO-66-NH_2_ as a support.

**Figure 5:**
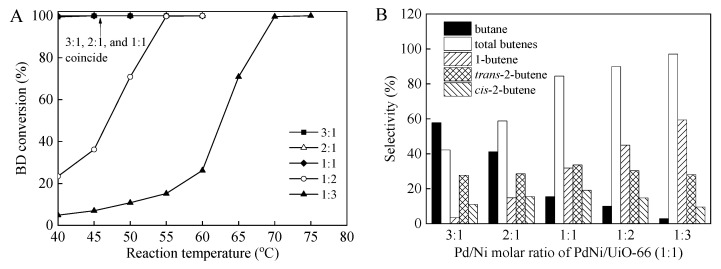
(**A**) BD conversions at different reaction temperature over PdNi/UiO-66 with diverse Pd/Ni molar ratios; (**B**) Product selectivities over PdNi/UiO-66 with diverse Pd/Ni molar ratios at nearly 100% BD conversion; (reaction conditions: 5 mg of catalyst, 6.5 mL/min of H_2_ flow rate, 20 mL/min of 1.0 vol.%BD/N_2_ flow rate. BD conversion and product selectivities given in (**A**,**B**) were stable data at reaction temperature).

**Figure 6:**
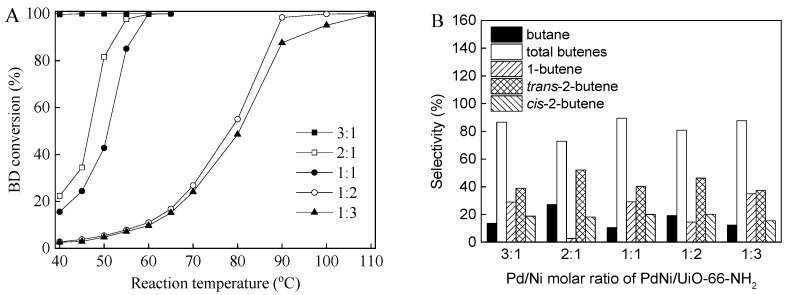
(**A**) BD conversions at different reaction temperature over PdNi/UiO-66-NH_2_ with diverse Pd/Ni molar ratios; (**B**) Product selectivities over PdNi/UiO-66-NH_2_ with diverse Pd/Ni molar ratios at nearly 100% BD conversion; (reaction conditions: 5 mg of catalyst, 6.5 mL/min of H_2_ flow rate, 20 mL/min of 1.0 vol.%BD/N_2_ flow rate. BD conversion and product selectivities given in (**A**,**B**) were stable data at reaction temperature).

The molar ratio of the bimetallic metals presented a remarkable effect on the catalytic activity and selectivity in the hydrogenation of BD, because it affects the surface composition of bimetallic supported catalysts [1,15]. The effects of the Pd/Ni molar ratio of the PdNi/UiO-66 and PdNi/UiO-66-NH_2_ catalysts on the BD conversions and product selectivities for the BD hydrogenation were investigated (Figure 5 and Figure 6). For all the catalysts, the BD conversions and selectivities were found to be dependent on the molar ratio of Pd to Ni. The PdNi/UiO-66 catalyst with Pd/Ni ratios of 1:1, 2:1, and 3:1 displayed excellent BD conversions, reaching 99.8%, 100%, and 99.5% at 40 °C for the PdNi/UiO-66 (1:1), PdNi/UiO-66 (2:1), and PdNi/UiO-66 (3:1), respectively (Figure 5A). The BD conversions of PdNi/UiO-66 (1:3) and PdNi/UiO-66 (1:2) gradually increased with the increase in temperature; they were 99.6% and 99.8% at 70 °C and 55 °C for PdNi/UiO-66 (1:3) and PdNi/UiO-66 (1:2), respectively (Figure 5A). The selectivities of total produced butenes increased from 42.2% to 97.1% as the Pd/Ni molar ratio decreased from 3:1 to 1:3 at nearly 100% BD conversion (Figure 5B). Among all the tested PdNi/UiO-66 with different molar ratios, PdNi/UiO-66 (1:1) displays the highest catalytic activity and butene selectivity toward BD hydrogenation. For PdNi/UiO-66-NH_2_, the catalytic activity (BD conversion) enhanced with the raise in the Pd/Ni molar ratio. PdNi/UiO-66-NH_2_ (3:1) displayed the highest catalytic activity. A nearly complete BD conversion (99.7%) was achieved at 40 °C (Figure 6A). BD conversions of 98.4%, 95.1%, 100%, and 97.7% were achieved on PdNi/UiO-66-NH_2_ (1:3), PdNi/UiO-66-NH_2_ (1:2), PdNi/UiO-66-NH_2_ (1:1), and PdNi/UiO-66-NH_2_ (2:1) at 100 °C, 90 °C, 60 °C, and 55 °C, respectively (Figure 6A). The selectivities of total butenes were 87.7%, 80.8%, 89.5%, 72.8%, and 86.5% at nearly 100% BD conversion on PdNi/UiO-66-NH_2_ (1:3), PdNi/UiO-66-NH_2_ (1:2), PdNi/UiO-66-NH_2_ (1:1), PdNi/UiO-66-NH_2_ (2:1), and PdNi/UiO-66-NH_2_ (3:1), respectively (Figure 6B). Although the PdNi/UiO-66-NH_2_ (1:1) catalyst displayed moderate catalytic activity, the excellent butene selectivity displayed that the PdNi/UiO-66-NH_2_ catalyst with a Pd/Ni molar ratio of 1:1 rendered its optimal proportion.

**Figure 7:**
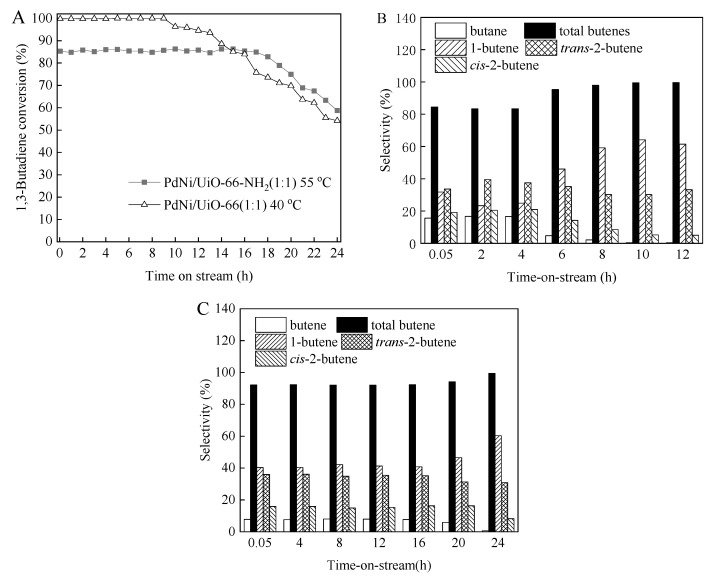
Evolution of the BD conversion and product selectivity with time-on-stream for PdNi/UiO-66 (1:1) and PdNi/UiO-66-NH_2_ (1:1) at 40 °C and 55 °C, respectively: (**A**) BD conversion; (**B**) Product selectivity for PdNi/UiO-66 (1:1); (**C**) Product selectivity for PdNi/UiO-66-NH_2_ (1:1) (reaction conditions: 5 mg of catalyst, 6.5 mL/min of H_2_ flow rate, 20 mL/min of 1.0 vol.%BD/N_2_ flow rate).

The durability of PdNi/UiO-66 (1:1) and PdNi/UiO-66-NH_2_ (1:1) was tested within a time on stream of 24 h at 40 °C and 55 °C, respectively. Figure 7 shows the time-on-stream effects on the BD conversion and product selectivity for PdNi/UiO-66 (1:1) and PdNi/UiO-66-NH_2_ (1:1). The initial BD conversions of 99.8% and 85.3% were obtained for PdNi/UiO-66 (1:1) and PdNi/UiO-66-NH_2_ (1:1), respectively (Figure 7A). For PdNi/UiO-66 (1:1), a steady BD conversion was obtained during the first 9 h time-on-stream. Then, the BD conversion decreases stepwise and the BD conversion was 54.2% after 24 h time-on-stream. The BD conversion remained constant for PdNi/UiO-66-NH_2_ (1:1) during the first 16 h time-on-stream, and then the BD conversion gradually decreased from 85.3% to 58.8% with further extension of the time-on-stream from 16 h to 24 h. The PdNi/UiO-66-NH_2_ (1:1) catalyst presented better stability than PdNi/UiO-66 (1:1) under the reaction conditions. This could be caused by the strong interaction between the –NH_2_ groups of UiO-66-NH_2_ and PdNi NPs. The selectivity to total butenes remained unchanged in the first 9 h and 16 h on PdNi/UiO-66 (1:1) and PdNi/UiO-66-NH_2_ (1:1), respectively (Figure 7B,C). Moreover, the butene selectivity slightly increased with extension of the time-on-stream (Figure 7B,C).

To evaluate the reproducibility of the catalyst, two batches of PdNi/UiO-66-NH_2_ (1:1) catalysts were synthesized by the impregnation method. The catalytic activity and selectivity of the two batches of catalysts for the hydrogenation of BD were investigated under the same conditions (Figure S6). Two batches of PdNi/UiO-66-NH_2_ (1:1) displayed similar BD conversions and product selectivities for the BD hydrogenation at 55 °C (Figure S6). PdNi/UiO-66-NH_2_ (1:1) catalyst displayed good reproducibility.

Pd/UiO-66 and Pd/UiO-66-NH_2_ presented a different catalytic property compared with Pd–Ni bimetallic catalysts (Figure S5). For Pd/UiO-66 catalyst, the conversion of BD was 90.2% at the initial stage of hydrogenation, and then quickly deactivated within 0.5 h. The BD conversion decreased to 13.9% at 50 °C for 12 h. However, there is a rise in selectivity to total butenes with increasing reaction time. The selectivity toward total butenes raised from 82.0% to 99.7% when the reaction time increased from 0.25 h to 8 h. Pd/UiO-66-NH_2_ catalyst presented complete conversion at the initial stage of hydrogenation. The BD conversions slightly decreased in the next 4 h, and then rapidly decreased in the next 8 h, while the selectivity to total butenes steadily increased with the increase of the reaction time. The BD conversion and butene selectivity were 52.4% and 95% at 50 °C for 12 h, respectively. Obviously, the Pd NPs supported on UiO-66-NH_2_ (100%) presented higher BD conversion than that supported on UiO-66 (90.2%). This result is contrary to those of Pd–Ni bimetallic catalysts. This may be explained by a previous study, which displayed that the catalysts with the smallest NP show higher BD conversion [31]. The TEM characterization shows that the Pd NP mean particle sizes were 14.6 nm and 7.7 nm for Pd/UiO-66 and Pd/UiO-66-NH_2,_ respectively_._ The catalytic activity for the hydrogenation of BD was dependent on both MOF support structure and particle size. Although the PdNi/UiO-66-NH_2_ (1:1) bimetallic catalyst (42.8% BD conversion at 50 °C) exhibited lower hydrogenation activity than Pd/UiO-66-NH_2_ (100% BD conversions at 50 °C), it showed higher butene selectivity than monometallic Pd catalysts (95.3% and 51.8%). The PdNi/UiO-66 (1:1) (100%) displayed higher BD conversions than Pd/UiO-66 (90.2%), while the butene selectivity of the former (69.7%) was lower than the latter (82%). EDS characterization presented that Pd and Ni components existed as individual NPs in PdNi/UiO-66-NH_2_ (1:1) and PdNi/UiO-66 (1:1) catalysts (Figure 2F and Figure 3F). The improvement in the catalytic activity and selectivity of PdNi/UiO-66 (1:1) and PdNi/UiO-66-NH_2_ (1:1) could be attributed to the synergistic effect between monometallic Pd NPs and Ni NPs. In addition, NH_2_-doping enhances the catalyst stability, and the Pd/UiO-66-NH_2_ (4 h) exhibits higher stability than Pd/UiO-66 (0.5 h). Compared with Pd/UiO-66-NH_2_ and Pd/UiO-66 catalysts, Pd–Ni bimetallic catalysts PdNi/UiO-66 (1:1) and PdNi/UiO-66-NH_2_ (1:1) displayed higher stability in the BD hydrogenation reaction, indicating that the interaction between Pd and Ni plays an important role in improving the stability of the catalyst.

## 4. Conclusions

A set of Pd–Ni or Pd catalysts supported on highly ordered MOFs of UiO-66 and UiO-66-NH_2_ was successfully synthesized. TEM and EDS characterization displayed that PdNi NPs or Pd NPs were substantially dispersed on the surface of UiO-66-NH_2_ and UiO-66. The interaction between –NH_2_ groups of UiO-66-NH_2_ and PdNi NPs or Pd NPs, as well as the interaction between Pd and Ni, can effectively prevent the aggregation of NPs. All catalysts were investigated in BD hydrogenation by using a fixed-bed, continuous-flow-type quartz reactor at 40–110 °C under ambient pressure. The results displayed that the structure of the MOF support, the molar ratio of Pd/Ni, and particle size have important effects on the catalytic activity and product selectivity. The UiO-66-NH_2_ with a certain number of alkaline sites supporting Pd–Ni bimetallic NP catalysts displayed a lower catalytic activity than that of UiO-66 in the selective BD hydrogenation. However, they were more favorable to the formation of butenes. NH_2_-doping and the synergistic interaction between Pd and Ni could enhance the catalyst stability. The bimetallic catalysts PdNi/UiO-66-NH_2_ (1:1) (16 h) and PdNi/UiO-66 (1:1) (9 h) displayed higher stability than Pd/UiO-66-NH_2_ (4 h) and Pd/UiO-66 (0.5 h). Moreover, the catalysts presented good reproducibility in the hydrogenation of BD. The bimetallic Pd–Ni catalysts with a Pd/Ni ratio of 1:1 demonstrated the best catalytic performance. These findings afford beneficial guidance for the design and preparation of high-efficiency catalysts for the selective hydrogenation of BD.

## Figures and Tables

**Table 1:** The textural properties of the samples.

Entry	Sample	BET (m^2^/g)	Mean Pore Diameter (nm)	Volume (cm^3^/g)
1	UiO-66	1467	2.1	0.75
2	PdNi/UiO-66 (1:1)	1292	2.2	0.70
3	UiO-66-NH_2_	1223	3.3	1.02
4	PdNi/UiO-66-NH_2_ (1:1)	851	3.8	0.81

**Table 2:** The BD conversion and butene selectivity toward total butenes over supported Pd-Ni bimetallic catalysts for the BD hydrogenation reaction.

Entry	Catalyst	Mean Particle Size (nm)	T (°C)	Conv. (%)	Sel. (%)	Lifetime (h)	Ref.
1	PdNi/UiO-66 (1:1)	7.1	40	99.8	84.5	9	This work
2	PdNi/UiO-66-NH_2_ (1:1)	4.6	60	100	89.5	16	This work
3	PdNi/γ-Al_2_O_3_	5.9	70	90	80	-	21
4	PdNi/SiO_2_	6.3	70	85	100	-	21
5	1NiPd/Al_2_O_3_	-	40	80	99.8	-	29
6	0.91%Pd1.51%Ni/γ-Al_2_O_3_	5.9	87	100	-	-	64

## Data Availability

The data presented in this study are available on request from the corresponding author.

## Supplementary Materials

The following supporting information can be downloaded at: https://www.mdpi.com/article/10.3390/nano12091484/s1, Figure S1: PXRD analysis of UiO-66, PdNi/UiO-66 (1:1), Pd/UiO-66, UiO-66-NH_2_, PdNi/UiO-66-NH_2_ (1:1), and Pd/UiO-66-NH_2_; Figure S2: The N_2_ adsorption-desorption isotherms (A) and pore size distribution (B,C) of UiO-66-NH_2_, PdNi/UiO-66-NH_2_(1:1), UiO-66, and PdNi/UiO-66(1:1); Figure S3: TEM images and Pd NP size distribution of Pd/UiO-66 (A,B) and PdNi/UiO-66-NH_2_ (C,D); Figure S4: XPS spectra of PdNi/UiO-66-NH_2_ (1:1) (A,B) and PdNi/UiO-66 (1:1) (C,D); Figure S5: Evolution of the BD conversion and product selectivity with time-on-stream for Pd/UiO-66 (A) and Pd/UiO-66-NH_2_ (B) at 50 °C; Figure S6: Evolution of the BD conversion and product selectivity with time-on-stream for the first batch and second batch of PdNi/UiO-66-NH_2_ (1:1) at 55 °C: (A) BD conversion; (B) Product selectivity for first batch; (C) Product selectivity for second batch; Table S1: The Pd and Ni weight content of Pd–Ni bimetallic catalysts.
