# Supplementary material for: Comparative Study of Pd–Ni Bimetallic Catalysts Supported on UiO-66 and UiO-66-NH2 in Selective 1,3-Butadiene Hydrogenation
Source: Nanomaterials (Basel). 2022 Apr 27;12(9):1484. doi: 10.3390/nano12091484 (PMC9105395; doi:10.3390/nano12091484)
Supplement: Supplementary file 1 [file nanomaterials-12-01484-s001.zip › nanomaterials-1695815-supplementary.pdf]

nanomaterials

Supplementary Materials

Comparative Study of Pd–Ni Bimetallic Catalysts Supported on UiO-66 and UiO-66-NH_2 in Selective 1,3-Butadiene Hydrogenation

Lili Liu [1], Lei Yu [1], Xiaojing Zhou [1], Chunling Xin [1], Songyuan Sun [1], Zhidong Liu [1], Jinyu Zhang [2], Ying Liu [1,*] and Xishi Tai [1,*]

[1] School of Chemistry & Chemical Engineering and Environmental Engineering, Weifang University, Weifang 261061, China; liulili122@wfu.edu.cn (L.L.); jimoyulei@163.com (L.Y.); zhouxiaojing105@wfu.edu.cn (X.Z.); 20150015@wfu.edu.cn (C.X.); ssy1210661650@foxmail.com (S.S.); liuzhidong0518@foxmail.com (Z.L.)
[2] Shandong Huazhiyuan Testing Co., Ltd., Weifang 261061, China; ziyjinyu@163.com
* Correspondence: liuying@wfu.edu.cn (Y.L.); taixs@wfu.edu.cn (X.T.)

Catalyst characterization

The structure of the samples was conducted on a X-ray diffractometer (XRD, Brüker D8 Advance, Karlsruhe, Germany) with a Ni filter Cu Kα (λ = 0.154 nm) radiation. The textural properties of the samples were investigated using a Quantachrome instrument (Boynton Beach, FL, USA) at 77 K after out-gassing at 150 °C for 12 h. The chemical valence of Pd and Ni was investigated using a PHI Quantum-2000 X-ray photoelectron spectrometer (XPS, Boynton Beach, FL, USA), with Al-Kα radiation of 1486.6 eV as incident beam. The morphology and Pd–Ni distribution were measured by a JEOL-JEM-2010 transmission electron microscopy (TEM, Jeol, Japan). Energy dispersive X-ray spectroscopy (EDS) was performed on an Oxford X-MaxN 80T IE250 instrument (Oxford, UK). The alkaline properties of UiO-66-NH_2 and UiO-66 were tested via CO_2 temperature-programmed desorption (CO_2-TPD) using Chemisorption Analyzer (MicrotracBEL AutoChem BELCAT-B, Bel, Osaka, Japan) equipped with a thermal conductivity detector. The Pd and Ni weight contents were studied using an Optima 5300DV inductively coupled plasma-optical emission spectrometer (ICP-OES, Waltham, MA, USA).

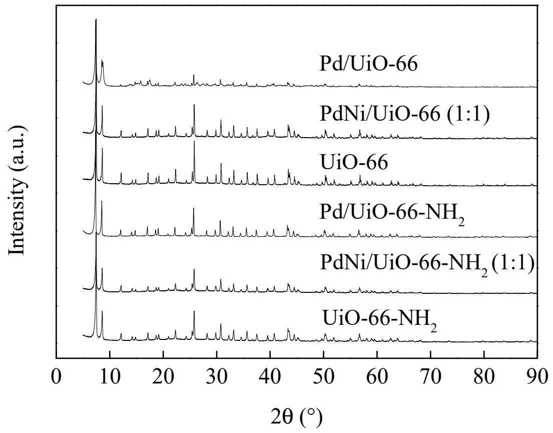

Figure S1. PXRD analysis of UiO-66, PdNi/UiO-66 (1:1), Pd/UiO-66, UiO-66-NH_2, PdNi/UiO-66-NH_2 (1:1), and Pd/UiO-66-NH_2.

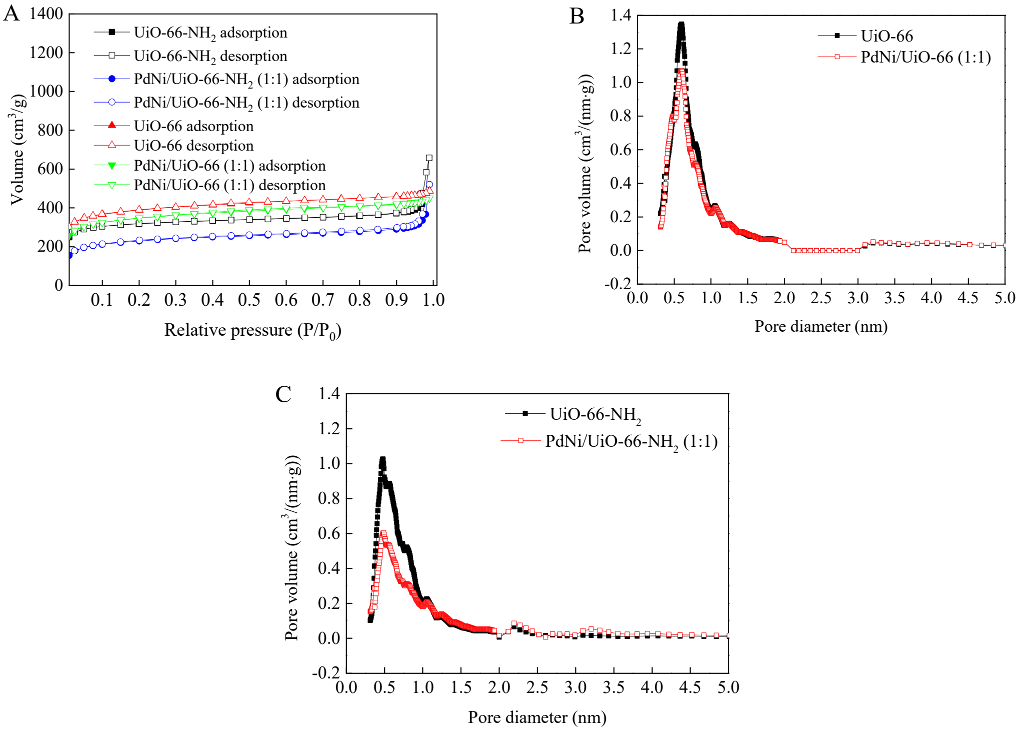

Figure S2. The N2 adsorption-desorption isotherms (**A**) and pore size distribution (**B**,**C**) of UiO-66-NH2, PdNi/UiO-66-NH2(1:1), UiO-66, and PdNi/UiO-66(1:1).

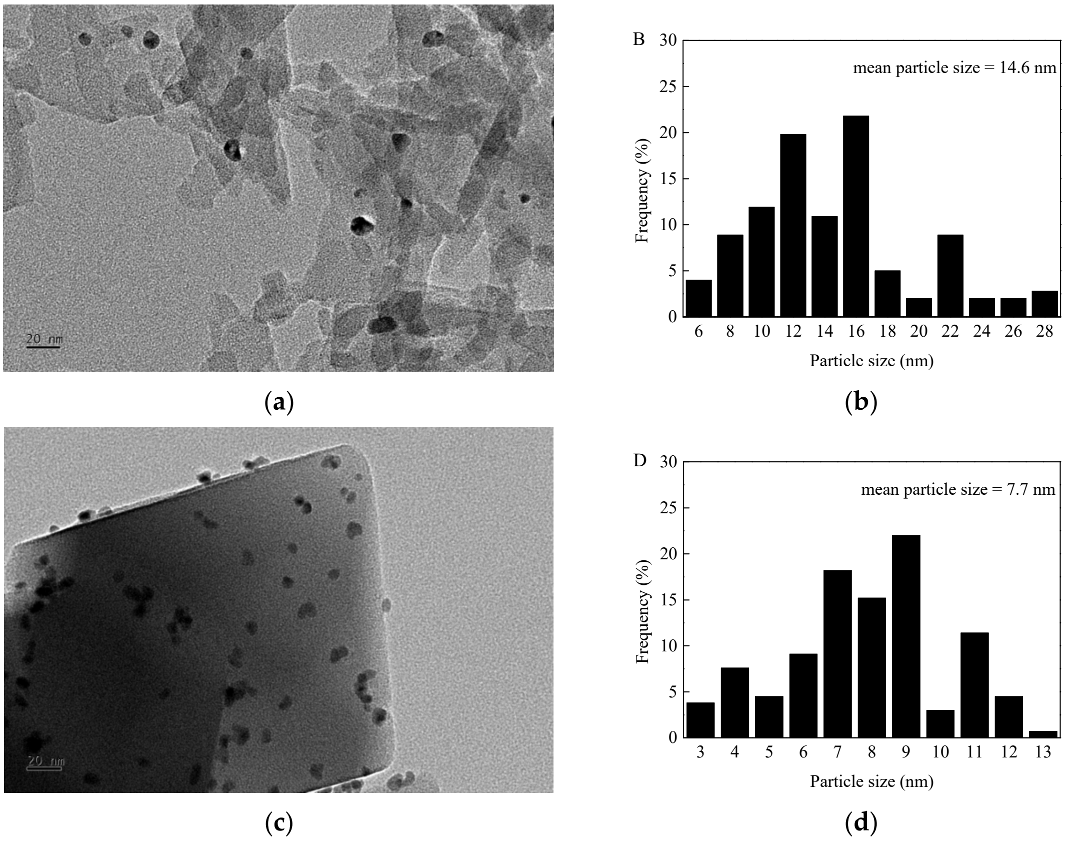

Figure S3. TEM images and Pd NP size distribution of Pd/UiO-66 (**A**,**B**) and Pd/UiO-66-NH2 (**C**,**D**).

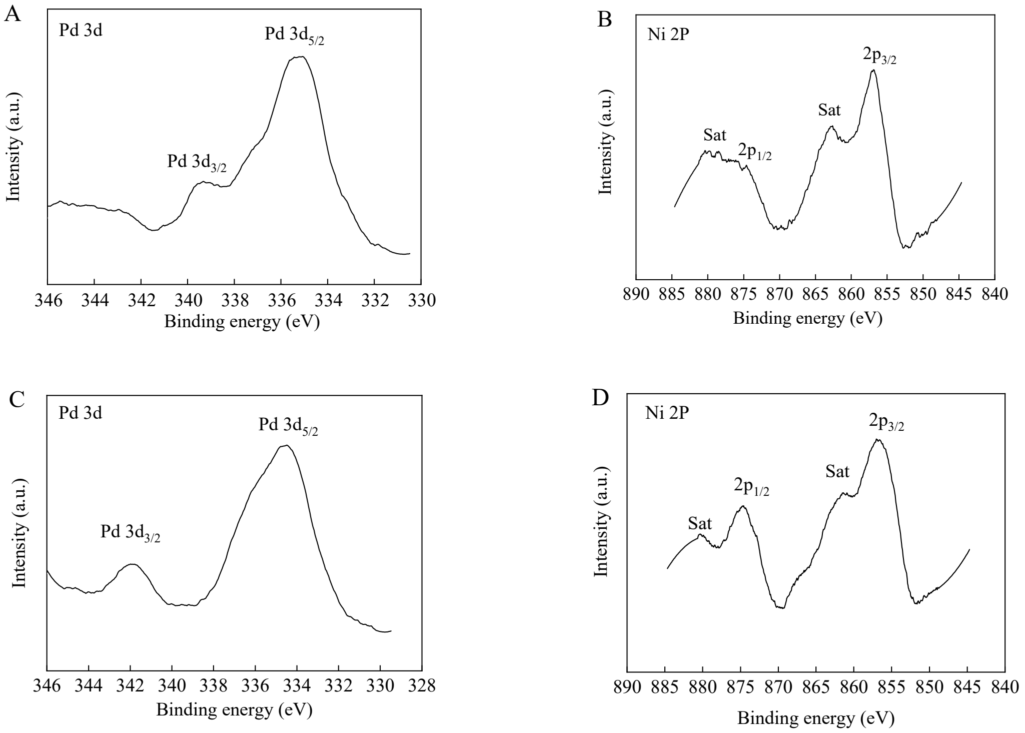

Figure S4. XPS spectra of PdNi/UiO-66-NH_2 (1:1) (**A**,**B**) and PdNi/UiO-66 (1:1) (**C**,**D**).

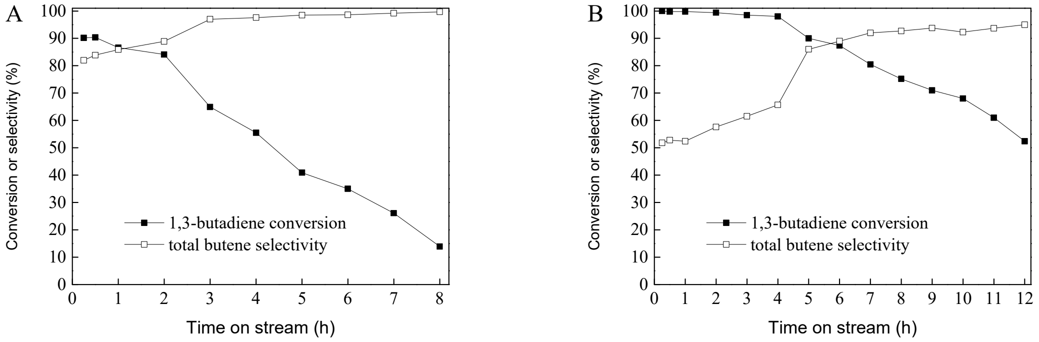

Figure S5. Evolution of the BD conversion and product selectivity with time-on-stream for Pd/UiO-66 (**A**) and Pd/UiO-66-NH_2 (**B**) at 50 °C (reaction conditions: 5 mg of catalyst, 6.5 mL/min of H_2 flow rate, 20 mL/min of 1.0 vol.%BD/N_2 flow rate).

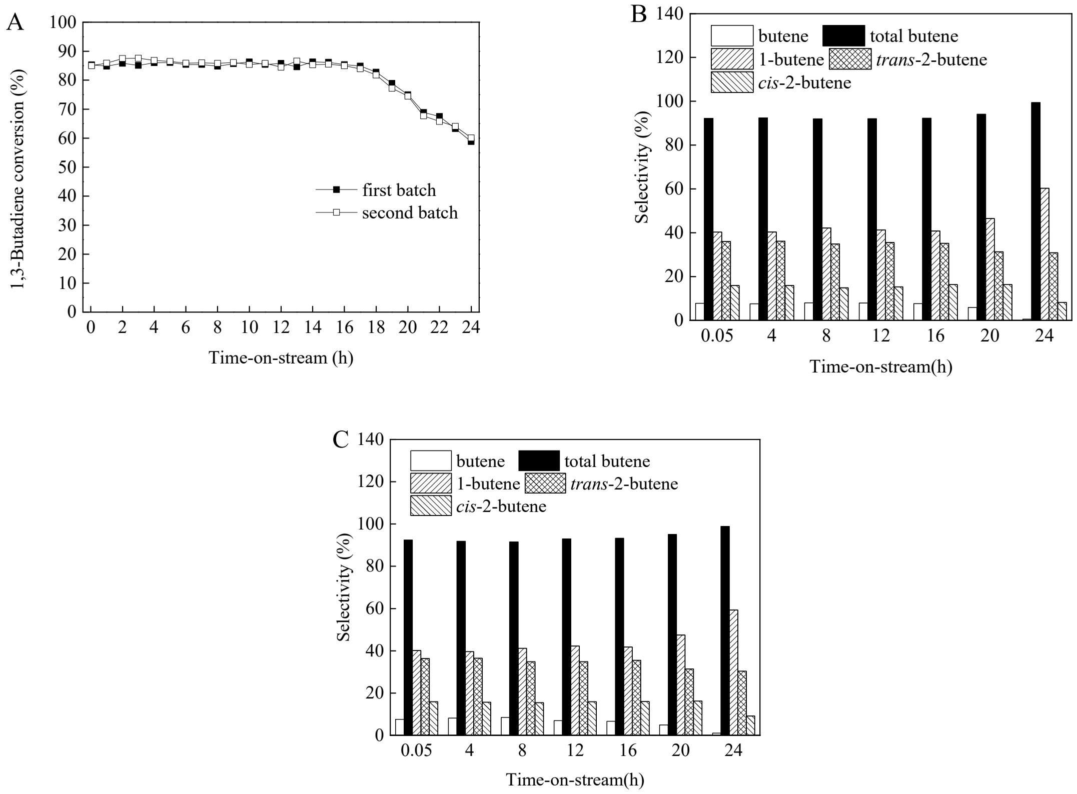

Figure S6. Evolution of the BD conversion and product selectivity with time-on-stream for the first batch and second batch of PdNi/UiO-66-NH_2 (1:1) at 55 °C: (**A**) BD conversion; (**B**) Product selectivity for first batch; (**C**) Product selectivity for second batch (reaction conditions: 5 mg of catalyst, 6.5 mL/min of H_2 flow rate, 20 mL/min of 1.0 vol.%BD/N_2 flow rate).

Table S1. The Pd and Ni weight content of Pd–Ni bimetallic catalysts.

Catalyst	Pd (wt%)	Ni (wt%)	Mole Ratio of Pd:Ni
PdNi/UiO-66-NH_2 (3:1)	3.31	0.65	3:1
PdNi/UiO-66-NH_2 (2:1)	2.93	0.85	2:1
PdNi/UiO-66-NH_2 (1:1)	2.51	1.33	1:1
PdNi/UiO-66-NH_2 (1:2)	1.79	2.12	1:2
PdNi/UiO-66-NH_2 (1:3)	1.49	2.55	1:3
PdNi/UiO-66 (3:1)	3.31	0.64	3:1
PdNi/UiO-66 (2:1)	2.92	0.94	2:1
PdNi/UiO-66 (1:1)	2.65	1.38	1:1
PdNi/UiO-66 (1:2)	1.82	2.19	1:2
PdNi/UiO-66 (1:3)	1.45	2.49	1:3
Pd/UiO-66-NH_2	1.54	-	-
Pd/UiO-66	1.42	-	-
